# Evolutionary Trees from the Tabloids and Beyond

**DOI:** 10.1007/s12052-010-0290-5

**Published:** 2010-10-08

**Authors:** Anastasia Thanukos

**Affiliations:** University of California Museum of Paleontology, 1101 Valley Life Sciences Building, Berkeley, CA 94720-4780 USA

**Keywords:** Phylogenetics, Applications, Teaching

In this special issue of *Evolution*: *Education and Outreach*, many authors (notably Brooks 2010) argue for the importance of helping students understand phylogenetics and outline innovative ways of introducing key concepts regarding tree reading (Mclennan 2010a) and tree building (Kumala 2010b). As argued by (Brooks 2010) and Kumala (2010a, c), evolutionary relationships (represented in phylogenies) can serve as the basic structure on which students hang their understanding of the biological world, providing a meaningful way to organize and remember facts, as well as serve as a constant reminder of the processes that have shaped biodiversity.

And of course, phylogenetics is important to understand in its own right. Biology has experienced something of a phylogenetic revolution in the last few decades (Losos 1996). Technology has advanced in several fields, making genetic sequences cheaper and faster to obtain and vastly improving our ability to analyze those data through increased computing power and new analytic methods. All of this has made phylogenies based on DNA easier to build and helped to highlight the importance of a phylogenetic perspective even when molecular data are unavailable (e.g., for most, but not all, fossil organisms). There’s a simple reason that phylogenies have become increasingly prevalent in textbooks and now even appear in middle school texts (Catley and Novick 2008): phylogenies have become increasingly important in biological research and in shaping how scientists look at the natural world. To grasp modern biology, students *must* understand the basics of phylogenetics.

Textbooks have responded to this need, as have the educators and scientists who develop supporting educational resources. For example, a typical high school textbook might use a phylogeny to illustrate the diversity of animal life, marking the evolution of key traits such as radial symmetry or the coelom. The same text might introduce the basics of reading phylogenies by explaining the concepts of common ancestry, clades, and shared derived characters. But how can students be motivated to learn and retain this material (i.e., not hit the delete button, which Mclennan (2010a) notes so frequently happens)? Using innovative and engaging teaching activities (e.g., Kumala 2010a) and narratives can help. In addition, instructors can incorporate examples of practical applications of phylogenetic reasoning that are compelling to students and relevant to their lives and basic social issues.

Here, we will focus on practical applications of phylogenetics, expanding on one example referenced in another article in this issue (Wiley 2010) and introducing selected additional examples ripe for deployment in classrooms. So what exactly can you do with a phylogeny? Lots...

## Catch a Killer

Perhaps the most widely circulated (and most tabloid-worthy) example of phylogenetics in action is the case of the State of Louisiana versus Richard J. Schmidt (Vogel 1996). As summarized by Wiley (2010), in 1995, Schmidt (a medical doctor) was accused of injecting his former mistress (Janet Allen, a nurse) with HIV-positive blood from one of his patients. Allen and Schmidt had been romantically involved for a decade, and Schmidt had been giving her regular vitamin injections. After threatening to break off the affair, Allen found that she was HIV positive and accused Schmidt of substituting tainted blood for one of her injections.

**Fig. 1:**
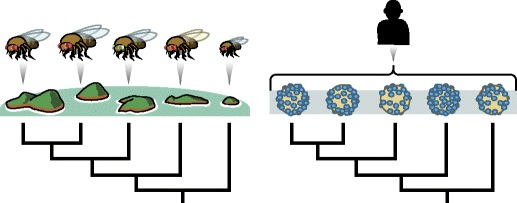
Fruit fly populations may evolve into separate species when separated geographically from one another for thousands of years, while HIV may evolve into separate lineages within a single individual in less than a year. Illustration adapted with permission from the Understanding Evolution website

Did the doctor do it? If Schmidt were accused of retaliating against Allen with a simple poison, the investigation would have taken a very different course. But unlike a poison, HIV has genetic material (RNA) and makes copies of itself, meaning that it can evolve. In fact, because of its high mutation rate and rapid replication rate, HIV evolves remarkably quickly. While the process of speciation may take tens or hundreds of thousands of years in animals like fruit flies, HIV can diversify into many different strains within a single individual in less than a year (Fig. 1). As HIV evolves, it accumulates mutations in its genome—some beneficial to the virus (and likely, bad for human hosts!), some slightly deleterious to the virus, and most with no notable effect at all. Whatever their impact, those mutations record events in the lineage’s history, turning the genome into something of a marked-up road map—a spotty, but informative, account of where the lineage has been at different points in its evolutionary past. This makes it possible for scientists to use the virus’s RNA sequence to reconstruct the family relationships among different strains (i.e., to build a phylogeny).

**Fig. 2:**
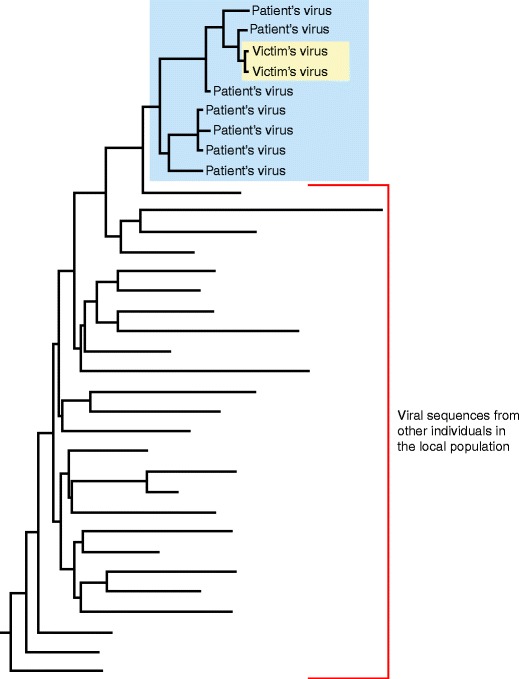
Phylogeny of HIV strains from the victim, the patient, and other locals, based on the sequence of the reverse transcriptase gene. The length of each branch indicates the degree of genetic divergence. Note that the victim’s viral branches are extremely short, indicating few differences from the patient’s sequences. Phylogeny based on Metzker et al. (2002)

Biologists compared samples of HIV strains from the victim to samples from the doctor’s HIV-positive patient and to viral strains from other HIV-positive people living in the local area (Metzker et al. 2002). The biologists sequenced different regions of the viral RNA and used these data to build phylogenies. Figure 2 shows the phylogeny they built using part of the HIV reverse transcriptase gene. Every HIV strain sequence is different, but the phylogeny shows how they are related. The victim’s sequences are most closely related to those of the doctor’s HIV-positive patient and are much more distantly related to other HIV strains. Even more convincingly, the victim’s sequences are nested within the clade formed by the patient’s sequences; they are a subset of the patient’s sequences. This is exactly what we would expect to observe if the patient was infected with HIV, the virus evolved into many different strains within him, and then the victim was infected with one of the patient’s strains, and the virus continued to evolve within her. Based partly on the strength of phylogenetic evidence such as this, the doctor was convicted of attempted murder in 1998 (Vogel 1996).

## Turn Back Time

Just as phylogenetic evidence can be used to convict a killer, it can also be used to exonerate the innocent—at least in theory. In 2004, six medical workers from Bulgaria were condemned to death in Libya where they had been working, convicted of deliberately infecting hundreds of hospitalized Libyan children with HIV (Butler 2006). Did they do it? Circumstances suggested that the Libyan authorities might have had the story wrong: many of the HIV-positive children were also infected with various hepatitis strains (suggesting repeated infection via dirty needles), and the hospital in question seemed to have unsafe medical practices.

**Fig. 3:**
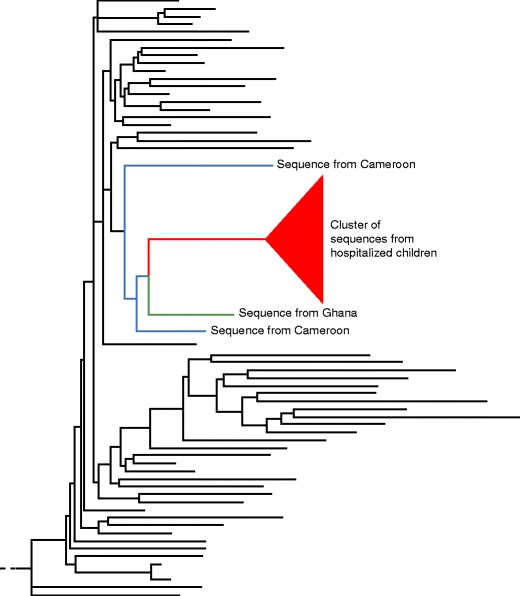
Phylogeny of HIV strains, including strains from the infected children. The length of each branch indicates the degree of genetic divergence. Phylogeny based on de Oliveira et al. (2006)

To answer the question, a team of biologists sequenced a particular HIV gene from the viruses infecting 44 of the children. As described above, HIV viruses evolve quickly because of their high mutation rates and short generation times—and their genetic sequences can be used to reconstruct that evolutionary history. The biologists compared the sequences from the children to many other known HIV sequences and used them to build a phylogeny (Fig. 3; de Oliveira et al. 2006). That tree revealed that the children’s viruses formed a tight-knit family—as would be expected if they stemmed from a single introduction of HIV to the hospital, which was then passed to many different patients. Based on the viral strains most closely related to the children’s (one strain from Ghana and two from Cameroon), the strain in question seems to have come from West Africa. Many West African migrants come to Libya seeking work. It seems likely that the virus was accidentally introduced to the hospital when an infected worker or a worker's infected child was treated there.

**Fig. 4:**
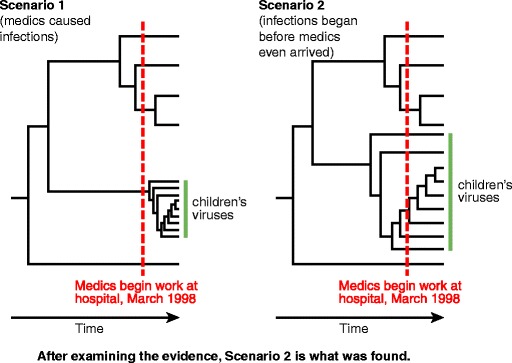
Hypothetical phylogenies showing how molecular clocks can be used to estimate the date of divergence of the children’s HIV virus strains. In Scenario 1, the children’s sequences show little divergence and so probably began diverging from one another after the medics’ arrival. In Scenario 2, the children’s sequences show substantial divergence and so probably began diverging from one another before the medics’ arrival. Illustration adapted with permission from the Understanding Evolution website

The biologists also used the amount of genetic change in the viruses to estimate when the different viral lineages split off from one another. This analysis was based on a molecular clock, the idea that in certain stretches of DNA or RNA, mutations accumulate at a reliable rate (e.g., one per year), allowing them to be used as timers: the more mutations the genetic material has accumulated, the longer since it split off from its ancestral sequence. The molecular clock analysis of the children’s HIV sequences convincingly showed that the medics could not have caused the disease cluster. The medics arrived at the hospital in March of 1998. If the medics *had* intentionally infected the children, the first victims would have been infected after the medics’ arrival—and hence, the victims’ viruses would have begun to diverge from one another and from other viral lineages *after* March of 1998 (as shown in Scenario 1, Fig. 4). But when the biologists traced the family tree of the children’s viruses back in time, that’s not what they found. Instead they discovered a phylogeny resembling that shown in Scenario 2 (Fig. 4); the children’s viruses were so different from one another that they must have begun diverging long before 1998. Children had been infected several years *before* the medics even arrived on the scene!

Unfortunately, even after this evidence was introduced in a retrial, the death sentence was upheld (Bohannon 2005). Finally, in 2007, after more than eight years of imprisonment, the medical workers were released and returned home (Bohannon 2007). However, their release did not come through the Libyan court system, which had repeatedly ignored scientific evidence supporting the medics’ innocence, but through political maneuvering and incentives: promises of aid, trade, debt write-offs, and payments to the infected children’s families.

## Identify Mystery Meat

While phylogenetic methods may indeed help your students identify the school cafeteria’s lunch special, the same techniques can also be used to tackle more pressing environmental issues. In Japan, whale meat is considered a delicacy—one that has become harder to find since global declines in whale populations spurred international agreements placing tight restrictions on which whales can be hunted, how they can be brought to market, and whether their meat can be imported and exported. Does the market for whale meat in Japan encourage illegal whaling and trading of whale products? The answer is hard to figure out simply by visiting Japanese fish markets. In these markets, purported whale is often simply labeled “whale” without specifying the meat’s species or provenance. Scott Baker and Steve Palumbi realized that phylogenetic analysis could help solve the problem.

**Fig. 5:**
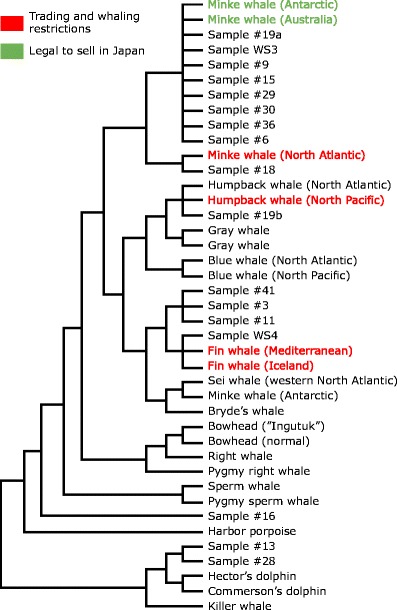
Phylogeny of mitochondrial DNA sequences from known whales and from “whale” meat bought at Japanese markets. Trading and whaling restrictions are only noted for whale species that appear to be closely related to a whale meat sample. Phylogeny based on Baker and Palumbi (1994)

The two scientists went to Japan in 1993, visited local markets, and bought many different samples of meat labeled “whale.” Because of restrictions on transporting whale tissues across international borders for scientific research, the American biologists performed much of the genetic grunt work for the analysis in their hotel rooms in Japan (Angier 1994). The researchers sequenced part of a mitochondrial gene from each meat sample, compared them to sequences from known whale populations, and used these data to build a phylogeny showing how all the sequences are related (Fig. 5; Baker and Palumbi 1994). Many of the sequences from the “whale” meat are closely related to sequences from whales that cannot be sold legally in Japan. That meat likely came from protected species, although the researchers dutifully noted that some of this meat might be considered legal if it had been kept in storage since before the restrictions were enacted—between four and 27 years! In addition, the analysis suggested that some of the “whale” meat could be dangerous to consumers; some meat appeared to come from dolphins, which eat higher on the food chain than baleen whales and so are more likely to contain dangerous levels of mercury.

## Choose Your Animal Companions Wisely

**Fig. 6:**
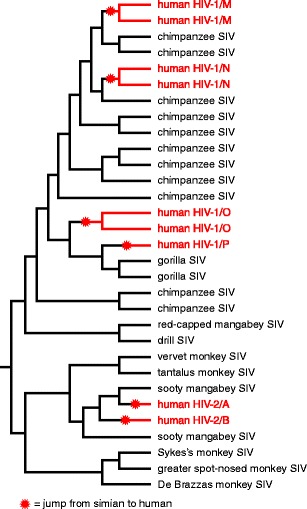
Phylogeny of HIV viruses and simian immunodeficiency viruses (SIV). Phylogeny based on Frontline (http://www.pbs.org/wgbh/pages/frontline/aids/) and Plantier et al. (2009) and adapted with permission from the Understanding Evolution website

Where do new pathogens come from? The obvious answer is “from old pathogens.” Phylogenetic analysis can help us figure out which old pathogens our new diseases came from—and such analyses have revealed that we are often the victims of inter-species germ swaps (Brooks and Hoberg 2008). Many of our so-called “new” diseases are simply old pathogens that used to infect other species but have recently evolved in ways that allow them to infect humans. HIV is perhaps the best-known example (as described in Wiley 2010), having made the leap to humans from simians many times (Fig. 6), but HIV is not alone. Ebola, West Nile Virus, and avian flu have all recently begun infecting humans as well.

In 2002 and 2003, when the airborne SARS virus caused 774 deaths, more than 8,000 cases of illness, and widespread panic, scientists and health workers alike wondered where it had come from (Normile 2005). In 2003, attention focused on cat-like mammals called civets because infected civets were discovered at a live animal market in southern China (where they are occasionally eaten and where SARS was a problem). However, further searches failed to turn up more tainted civets, suggesting that these animals were not the original source of the virus. Then in 2005, two teams of researchers independently discovered large reservoirs of a SARS-like virus in Chinese horseshoe bats. Could bats have been the original source of SARS? Figuring out the answer required reconstructing the evolutionary history of the virus.

**Fig. 7:**
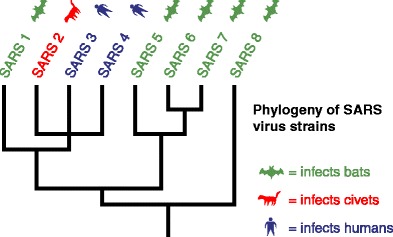
Phylogeny of a SARS virus gene, with hosts to different strains noted. Phylogeny based on Li et al. (2005) and reproduced with permission from the Understanding Evolution website

Biologists collected samples of the SARS virus’s RNA from different sources (infected humans, infected civets, and several species of infected horseshoe bat) and sequenced parts of their genomes (Li et al. 2005). They used these data to reconstruct the evolutionary relationship among the different strains (Fig. 7). The tree showed that the civet and human strains are very similar and, most importantly, that both are nested within a clade of bat viruses. This suggests that the ancestor of the civet and human strains was a bat virus! Based on this evidence, biologists came up with a plausible explanation: infected bats and uninfected civets came into contact at a market, the virus was transmitted to civets and then multiplied and evolved in civets in the public market, until eventually the virus hopped to humans—perhaps from civets.

Viruses make the jump from bats to human hosts frequently. In fact, they appear to be the natural reservoirs for many human viruses, including the Ebola, Hendra, and Nipah viruses as well as SARS. What is it about bats that makes them such a breeding ground for human viruses? Biologists aren’t sure, but they have some ideas. Bats’ tendency to roost in tightly packed caves with other bat species might encourage the transmission of viruses between species and provide opportunities for viruses to evolve and recombine with each other. Some of the new viral strains that result may be poised to move to other animals, including us!

## Save the Earth

In another article in this issue, Brooks and Mclennan (2010) argue convincingly that our conservation goals should be broad—to save as many species and habitats as we possibly can, in circumstances that will allow the organisms to continue to evolve. Unfortunately, though we may aim to maintain this goal, we can’t save everything. Resources to direct towards conservation efforts are limited, and sometimes, difficult decisions must be made. But how do we make those choices? Many biologists have suggested that phylogenetic analysis can help in this process (e.g., Vane-Wright et al. 1991; Crozier 1997).

**Fig. 8:**
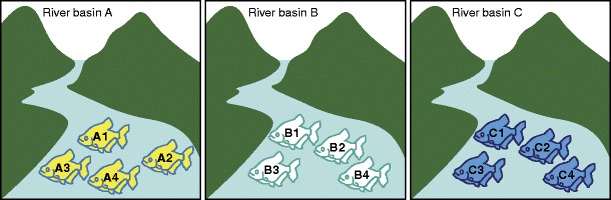
Hypothetical river basins with fish species noted. Illustration reproduced with permission from the Understanding Evolution website

**Fig. 9:**
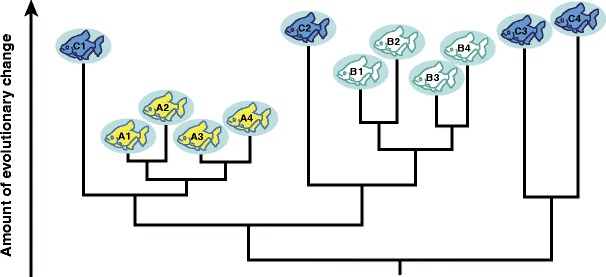
Hypothetical phylogeny of fish species. Illustration reproduced with permission from the Understanding Evolution website

The basic idea is that—although many other important considerations are involved in making these decisions—we should aim to prioritize conserving ecosystems and sets of species that preserve the greatest amount of evolutionary history. As a simplified example, imagine that a federal agency only has the resources to create a preserve in one of three river basins (Fig. 8). Each river basin happens to support four related fish species. If we want to conserve biodiversity and focus simply on the number of fish species, there is little difference between the three rivers. Now imagine that we reconstruct the evolutionary relationships among fish and build the phylogeny shown in Fig. 9. Even though each basin contains the same number of fish species, basin C includes more distantly related species. If we focus conservation efforts on basin C, we will preserve more evolutionary history and likely more genetic variation and evolutionary potential for the future.

Biologists recently put some of these ideas to the test (Cadotte et al. 2008). They wondered if some plants might be more important than others in preserving a functional ecosystem. They reasoned that the biomass produced by plants might be a particularly important indicator of a diverse, functioning ecosystem. After all, more biomass translates into more plant mass providing food for animals, producing oxygen, and absorbing the greenhouse gas carbon dioxide. Furthermore, the scientists suspected that the evolutionary relationships among an ecosystem’s plants help determine the amount of biomass they can produce. An ecosystem based on distantly related plants might be more productive than one based on closely related plants, they reasoned, since the distant relatives are more likely to have evolved to occupy distinct niches.

**Fig. 10:**
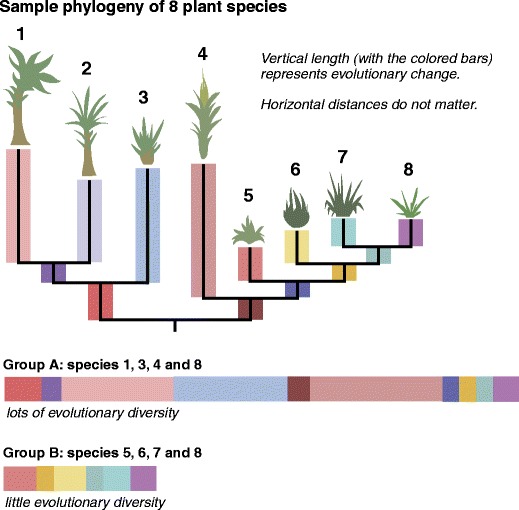
Hypothetical phylogeny of plant species with illustration of the total evolutionary diversity of two different sets of plants. Illustration reproduced with permission from the Understanding Evolution website

The biologists used phylogenetics to estimate the evolutionary distance between sets of plants. Figure 10 shows a hypothetical example. In this phylogeny, the length of each branch shows the amount of evolutionary change that occurred along that branch. Long branches mean lots of evolution; short branches mean little change. We can map a group of organisms onto one of these phylogenies and add up the lengths of all the branches that connect them in order to estimate the amount of evolutionary history encompassed by that group of organisms. In this example, you can see that group A has a much greater breadth of evolutionary history than does group B.

**Fig. 11:**
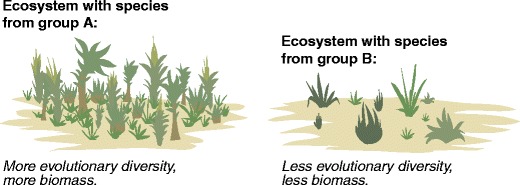
Ecosystems populated with organisms of greater evolutionary diversity produce more biomass. Illustration reproduced with permission from the Understanding Evolution website

The researchers studied 29 experiments that compared the biomass produced by different groups of plants. Their findings showed that evolutionary diversity makes a difference! Ecosystems with more diverse groups of plants (i.e., plants spanning more of the tree of life, as shown by group A, Fig. 10) tend to produce more biomass (Fig. 11). This finding supports the idea that, if we want to preserve functioning ecosystems, we should prioritize conserving evolutionarily distant species. For example, if forced to choose, we may want to put our resources into protecting species like the buttercup, which occupies a longer branch on the tree of life than its closely related grassland neighbors, sunflowers, and daisies.

## Conclusions

Here, we’ve outlined and illustrated just a few of the more engaging examples of phylogenetics in action in the real world. But there are many more examples of practical and scientific applications of phylogenetics—for example, in classification (as discussed in Mclennan 2010a; Wiley 2010; and Thanukos 2009) and in testing hypotheses about evolution (e.g., see Mclennan 2010b for examples of testing hypotheses about human behavior). Incorporating examples such as these into instruction on evolution can help students view phylogenetics as more than a complicated method of analysis practiced by biologists. It can encourage them to see phylogenetics and evolutionary relationships as a useful lens through which any biological problem—from the mundane, to the sensational, to the weighty—can be viewed.

## Give Me an Example of That

Want additional examples of how phylogenies can be used both in scientific research and to solve practical problems? Check out this tutorial from the Understanding Evolution website:

- Using trees. Find out how scientists use trees to make predictions about fossils, to learn about the evolution of complex features, to make predictions about poorly studied species, to learn about the order of evolution, and to learn about the evolution of diversity. Read it at: http://evolution.berkeley.edu/evolibrary/article/_0_0/phylogenetics_09

## Branch Out

In this article, we focused on using phylogenetics to solve practical problems; however, it’s important to keep in mind that phylogenetics is key in answering all sorts of scientific questions as well. Find out how trees can help us test hypotheses about evolutionary history:

- Using trees to understand plants—a research profile that follows scientist Chelsea Specht as she pieces together the evolutionary history of tropical plants and their pollinators—and in the process, tries to figure out how to conserve endangered species. http://evolution.berkeley.edu/evolibrary/article/specht_01

Find out how trees can be useful in biological classification:

- Using trees for classification—a brief tutorial that reviews the basics of phylogenetic classification. http://evolution.berkeley.edu/evolibrary/article/phylogenetics_04
- The new shrew that’s not—a news brief that describes scientists’ discovery of a new mammal species, a giant elephant shrew, and how this animal was classified. http://evolution.berkeley.edu/evolibrary/news/080301_elephantshrew
- A name by any other tree—an article on phylogenetic classification from a previous issue of this journal. http://www.springerlink.com/content/k176638503p63017/

## Dig Deeper

To dig deeper into some of the examples discussed in this article, visit the following Understanding Evolution resources:

- Evolutionary evidence takes the stand. This news brief describes the role of phylogenetic evidence in a Libyan court case. Six medical workers have been convicted of injecting children with HIV-tainted blood—but the evolutionary history of the virus paints a different picture. http://evolution.berkeley.edu/evolibrary/news/070101_libya
- Tracking SARS back to its source. This news brief traces the source of the SARS virus. Using phylogenetics, biologists have come up with a plausible path of transmission which may help us prevent future outbreaks of diseases such as HIV, SARS, and West Nile virus. http://evolution.berkeley.edu/evolibrary/news/060101_batsars
- Tough conservation choices? Ask evolution. The Earth is facing a biodiversity crisis. Nearly 50% of animal and plant species could disappear within our lifetime. To stem this rapid loss of biodiversity, we’ll need to act quickly, but where should we begin? This news brief explains how evolutionary history can help us set conservation priorities. http://evolution.berkeley.edu/evolibrary/news/081201_phylogeneticconservation

## In the Classroom

Before students can grasp the applications of phylogenetics, they’ll need to understand the basics of evolutionary trees. To build students’ tree-thinking skills, check out the other articles in this special issue, and try the following activities:

- What did T. Rex taste like? In this web-based module for grades 6–12 from the UC Museum of Paleontology, students are introduced to cladistics, which organizes living things by common ancestry and evolutionary relationships. http://www.ucmp.berkeley.edu/education/explorations/tours/Trex/index.html
- Nuts and bolts classification: arbitrary or not? In this lesson for grades 6–12 from the Evolution and the Nature of Science Institute, students working in teams classify furniture, share their categories and rationales, then note how their different schemes are perfectly logical and useful, but they vary and are completely arbitrary. They then see how living organisms are classified, and note how these natural groupings reflect the same ancestral relationships in the same nested hierarchies, regardless of the different criteria used. This concept is exemplified using primate phylogenetic trees. http://www.indiana.edu/~ensiweb/lessons/cl.intro.html
- Classification and Evolution. In this lesson for grades 9–12 from Robert Gendron, students construct an evolutionary tree of imaginary animals (Caminalcules) to illustrate how modern classification schemes attempt to reflect evolutionary history. http://nsm1.nsm.iup.edu/rgendron/labs.shtml

Once students understand what trees represent and feel confident reading them, you can introduce examples of phylogenetics in action. Try incorporating the examples discussed in this article into your classroom discussion or assigning students some of the readings listed above in Dig Deeper:

- http://evolution.berkeley.edu/evolibrary/news/070101_libya
- http://evolution.berkeley.edu/evolibrary/news/060101_batsars
- http://evolution.berkeley.edu/evolibrary/news/081201_phylogeneticconservation

Be sure to use the links to background material and the discussion/homework questions included with the readings. To introduce the Mystery Meat example, have students try this online activity:

- http://www.paleobio.org/MysteryMeat/

For undergraduates or advanced students, more challenging assignments may be appropriate:

- Ask students to compare and contrast the phylogenetic reasoning used in the Catch a Killer and Mystery Meat examples to the phylogenetic reasoning underlying the Turn Back Time example.
- Ask students to compare and contrast the phylogenetic reasoning used in the Turn Back Time example to that used in the story HIV’s Not-so-ancient History (http://evolution.berkeley.edu/evolibrary/news/081101_hivorigins).
- Divide students into small groups and challenge each group to identify a case of phylogenetic reasoning in action and prepare a short presentation for the class.

## References

[CR1] Angier N. DNA tests find meat of endangered whales for sale in Japan. NY Times 13 September 1994. http://www.nytimes.com/1994/09/13/science/dna-tests-find-meat-of-endangered-whales-for-sale-in-japan.html. Accessed September 8, 2010

[CR2] Baker CS, Palumbi SR (1994). Which whales are hunted? A molecular genetic approach to monitoring whaling. Science.

[CR3] Bohannon J (2005). Evidence overruled: medics on death row. Science.

[CR4] Bohannon J. Libya frees foreign medical workers. Science Now 24 July 2007. http://news.sciencemag.org/sciencenow/2007/07/24-02.html. Accessed September 8, 2010

[CR5] Brooks DR. Sagas of the children of time: the importance of phylogenetic teaching in biology. Evol Educ Outreach 2010;4. doi:10.1007/s12052-010-0268-3.

[CR6] Brooks DR, Hoberg EP (2008). Darwin’s necessary misfit and the sloshing bucket: the evolutionary biology of emerging infectious diseases. Evol Educ Outreach.

[CR7] Brooks DR, Mclennan DA. The biodiversity crisis: lessons from phylogenetic sagas. Evol Educ Outreach 2010;4. doi:10.1007/s12052-010-0269-2.

[CR8] Butler D (2006). Lawyers call for science to clear AIDS nurses in Bulgaria. Nature.

[CR9] Cadotte MW, Cardinale BJ, Oakley TH (2008). Evolutionary history and the effect of biodiversity on plant productivity. PNAS USA.

[CR10] Catley KM, Novick LR (2008). Seeing the world for the trees: an analysis of evolutionary diagrams in biology textbooks. Bioscience.

[CR11] Crozier RH (1997). Preserving the information content of species: genetic diversity, phylogeny, and conservation worth. Annu Rev Ecol Syst.

[CR12] de Oliveira T, Pybus OG, Rambaut A, Salemi M, Cassol S, Ciccozzi M (2006). HIV-1 and HCV sequences from Libyan outbreak. Nature.

[CR13] Kumala M. A natural history of you. Evol Educ Outreach 2010a;4. doi:10.1007/s12052-010-0276-3.

[CR14] Kumala M. The gummy tree challenge—building connections one treat at a time. Evol Educ Outreach 2010b;4. doi:10.1007/s12052-010-0275-4.

[CR15] Kumala M. The neverending story: using the narrative as a fundamental approach to teaching biology and beyond. Evol Educ Outreach 2010c;4. doi:10.1007/s12052-010-0277-2.

[CR16] Li W, Shi Z, Yu M, Ren W, Smith C, Epstein JH (2005). Bats are natural reservoirs of SARS-like coronaviruses. Science.

[CR17] Losos J (1996). Phylogenies and comparative biology, stage II: testing causal hypotheses derived from phylogenies with data from extant taxa. Syst Biol.

[CR18] Mclennan DA. How to read a phylogenetic tree. Evol Educ Outreach 2010a;4. doi:10.1007/s12052-010-0273-6.

[CR19] Mclennan DA. Sociobiology and the comparative approach: one way to study ourselves. Evol Educ Outreach 2010b;4. doi:10.1007/s12052-010-0274-5.

[CR20] Metzker ML, Mindell DP, Liu X, Ptak RG, Gibbs RA, Hillis DM (2002). Molecular evidence of HIV-1 transmission in a criminal case. PNAS USA.

[CR21] Normile D (2005). Researchers tie deadly SARS virus to bats. Science.

[CR22] Plantier J, Leoz M, Dickerson JE, de Oliveira F, Cordonnier F, Lemée V (2009). A new human immunodeficiency virus derived from gorillas. Nat Med.

[CR23] Thanukos A (2009). A name by any other tree. Evol Educ Outreach.

[CR24] Vane-Wright RI, Humphries CJ, Williams PH (1991). What to protect—systematics and the agony of choice. Biol Conserv.

[CR25] Vogel G (1996). HIV strain analysis debuts in murder trial. Nature.

[CR26] Wiley EO. Why trees are important. Evol Educ Outreach 2010;4. doi:10.1007/s12052-010-0279-0.

